# Supplementary material for: Molecular Characterization of Sterol C4-Methyl Oxidase in Leishmania major
Source: Int J Mol Sci. 2024 Oct 10;25(20):10908. doi: 10.3390/ijms252010908 (PMC11507432; doi:10.3390/ijms252010908)

```
L. major      1 MVFDTDFFVVNCSQMAANVLQSYIVAALHSAATQLPSSVQPYAMMLTRED     50
                              ||||.|.|:||||||:|||:||||||||||:|||||
L. donovani   1 --------------MAANALHSHIVAALHNAATKLPSSVQPYAMLLTRED     36

L. major     51 MVSTTLATAIATAVILYTVITVVLPVLRMDFYLSKLPTIKNSIPFLGHAL    100
                |||||||||||||||||||||||||||||||||||||||||.||||||||
L. donovani  37 MVSTTLATAIATAVILYTVITVVLPVLRMDFYLSKLPTIKNGIPFLGHAL     86

L. major    101 LLAGPSPWSKMSNWSLYPEKNLPQKKKSVDGPQTSRLVTYNVAGMRVIYI    150
                ||||||||||||||||||||||||||||.|||||||||||||||||||||
L. donovani  87 LLAGPSPWSKMSNWSLYPEKNLPQKKKSADGPQTSRLVTYNVAGMRVIYI    136

L. major    151 NEPRLLRRVLLTHQRNYRKALAAAYKHFMCLLGTGLVTSEDEQWKKGRLL    200
                ||||||||||||||||||||||||||||||||||||||||||||||||||
L. donovani 137 NEPRLLRRVLLTHQRNYRKALAAAYKHFMCLLGTGLVTSEDEQWKKGRLL    186

L. major    201 LSHAMRIDILDSVPEMAMKAVDRILLKLDAVDAKNPSVDLNEEYRHMTLQ    250
                ||||||||||||||||||||||||||||||||||||||||||||||||||
L. donovani 187 LSHAMRIDILDSVPEMAMKAVDRILLKLDAVDAKNPSVDLNEEYRHMTLQ    236

L. major    251 VISESALSLSAEESDRIFPALYLPIVHECNKRVWAPWRAYMPFLHGSRMR    300
                ||||||||||||||||||||||||||||||||||||||||||||.|||:|
L. donovani 237 VISESALSLSAEESDRIFPALYLPIVHECNKRVWAPWRAYMPFLQGSRVR    286

L. major    301 NRCLSELNKVLRDIICRRWEQRNDSNYTAKPDILALCISQIDRIDEKMIV    350
                |.||||||||||:|||||||||||||.|.||||||||||||||:||||||
L. donovani 287 NHCLSELNKVLRNIICRRWEQRNDSNCTGKPDILALCISQIDRMDEKMIV    336

L. major    351 GLIDDVKTILLAGHETSAALLTFATYEVLRHPEIRQKILEEATRLFDPAR    400
                ||||||||||||||||||||||||||||||||||||||||||||||||||
L. donovani 337 GLIDDVKTILLAGHETSAALLTFATYEVLRHPEIRQKILEEATRLFDPAR    386

L. major    401 CTCTVQTRYGPRGVPALNDVRSLVWTPAVLRETLRRHSVVPLVMRYAAKD    450
                ||.|||||||||||||:||||.||||||||||||||||||||||||||||
L. donovani 387 CTRTVQTRYGPRGVPAVNDVRDLVWTPAVLRETLRRHSVVPLVMRYAAKD    436

L. major    451 DVWPAADTGLDADVRIPAGCTIAVGIEGVHNNPDVWNKPEVFDPTRFIDA    500
                ||||||||||||||||||||||||||||||||||||||||||||||||||
L. donovani 437 DVWPAADTGLDADVRIPAGCTIAVGIEGVHNNPDVWNKPEVFDPTRFIDA    486

L. major    501 EIANDTNYLNQSTKDVKFAKKIDPYAFIPFINGPRNCLGQHLSMIETQVA    550
                ||||||||||||||||||||||||||||||||||||||||||||||||||
L. donovani 487 EIANDTNYLNQSTKDVKFAKKIDPYAFIPFINGPRNCLGQHLSMIETQVA    536

L. major    551 LAYMVLSYDLTIYRDPSYKGDVAAYEDAVGRHHDFIIPQVPHDGLKVWGT    600
                ||||||:||||||||||||||.||||||||||||||||||||||||||||
L. donovani 537 LAYMVLNYDLTIYRDPSYKGDAAAYEDAVGRHHDFIIPQVPHDGLKVWGT    586

L. major    601 PNKLFM    606
                ||||||
L. donovani 587 PNKLFM    592
```

Figure S1. Pair-wise alignment of *L. major* CYP5122A (LmjF.27.0090) and *L. donovani* CYP5122A1 (LdBPK_270090.1) using EMBOSS Needle. Identity: 94.4%. Similarity: 95.9%.

```
Lm      --------------------MV---LAKKQMKIPPIHKLRPGWSAVALHTVIYLLRHDFF     37
At      ------------------------------------------------------------     0
Sc      MSAVFNNATLSGLVQASTYSQTLQNVAHYQPQLNFMEKYWAAWYSYM-------------     47
Mm      MA---TNK-SVGVFS--SASLAVEYVDSLLPENPLQEPFKNAWVYML-------------     41

Lm      TAIVATSIVQPLYHRLILQNTYLQRLSDP--ALFTLVFAVLCHCVPWAFFNGIFLFFDSI     95
At      -----------------------------------------MLLLPFMVN---TYFS---     13
Sc      -------------------NNDV--LATGLMFFLLHEFMYFFRCLPWFIIDQIPYFR-R-     84
Mm      -------------------DNYTKFQIATWGSLIVHEAIYFLFSLPGFLFQFIPYMR-K-     80
                                                    :*  .      :

Lm      HPQYGIEGLRNNALLAPLGRRMAAYKLPRKPQQLPSAALIFSTMLHTAINHYLIIPVVLY     155
At      ------------------------FVPM--QTKNNTPAAQGKCITRLLLYHFSVNLPLML     47
Sc      ------------------------WKLQ--PTKIPSAKEQLYCLKSVLLSHFLVEAIPIW     118
Mm      ------------------------YKIQ--KDKPETFEGQWKCLKKILFNHFFIQLPLIC     114
                                :       :  :       :    : *: :    :

Lm      AYLVHTNSCALRAPPPESAIVGFAPEDVVNYMSGSNLRQIPISLVTITSHFLIANVINEM     215
At      ASYPVFRA------------MG----------MRSSFP-LPSW-KEVSAQILFYFIIEDF     83
Sc      TFHPMCEK------------LG----------ITVEVP-FPSL-KTMALEIGLFFVLEDT     154
Mm      GTYYFTEF------------FN----------IPYDWERMPRWYLTLAR-CLGCAVIEDT     151
              .             ..             .   :*     ::       ::::

Lm      GFYIVHSMLHSSPTLYRVFHKKHHMYTGTISIAAEYATPLEGILANAIPTTAYFTFMFFH     275
At      VFYWGHRILHS-KWLYKNVHSVHHEYATPFGLTSEYAHPAEILFLGFATIVGPAL-----     137
Sc      WHYWAHRLFHY-GVFYKYIHKQHHRYAAPFGLSAEYAHPAETLSLGFGTVGMPILY--VM     211
Mm      WHYFLHRLLHH-KRIYKYIHKVHHEFQAPFGIEAEYAHPLETLILGTGFFIGIVL-----     205
         .*  * ::*    :*: .*. ** :   :.: :*** * * :  .

Lm      YTREEASKSSFVTSARAWPLFITWMWARLWETYEVHSGYCFSDTWLGKLGLLHGHRARFH     335
At      -------------TGPHLITLWLWMVLRVLETVEAHCGYHFPWSLSNFLPLYG--GADFH     182
Sc      Y-----------TGKLHLFTLCVWITLRLFQAVDSHSGYDFPWSLNKIMPFWA--GAEHH     258
Mm      -------------LCDHVILLWAWVTIRLLETIDVHSGYDIPLNPLNLVPFYT--GARHH     250
                            :  *:  *: :: : *.** :  .    : :     * .*

Lm      DFHHTHN---VCNYGSGL-FMDALLNTMDPYLIYRYPDKHPHTTALKEEDLKEPRDLEVG     391
At      DYHHRLLYTKSGNYSSTFVYMDWIFGTDKGYRRL----------KTLKENG---------     223
Sc      DLHHHYF---IGNYASSFRWWDYCLDTESGPEAK----------ASREERMKKRAEN---     302
Mm      DFHHMNF---IGNYASTFTWWDKLFGTDAQYHAY----------IEKSKKLGKKSD----     293
        * **        **.* : : *  :.*                    .:

Lm      SEAMDQVRACS     402
At      --DMKQT----     228
Sc      -NAQKKTN---     309
Mm      -----------     293
```

Figure S2. The alignment of ERG25 proteins (Lm: *L. major*; At: *Arabidopsis thaliana*; Sc: *Saccharomyces cerevisiae*; Mm: *Mus musculus*) using Clustal Omega. Asterisks (*): fully conserved residues; colons (:): highly similar residues; periods (.): weakly similar residues. *L. major* ERG25 shows 23-25% identity to other ERG25s.

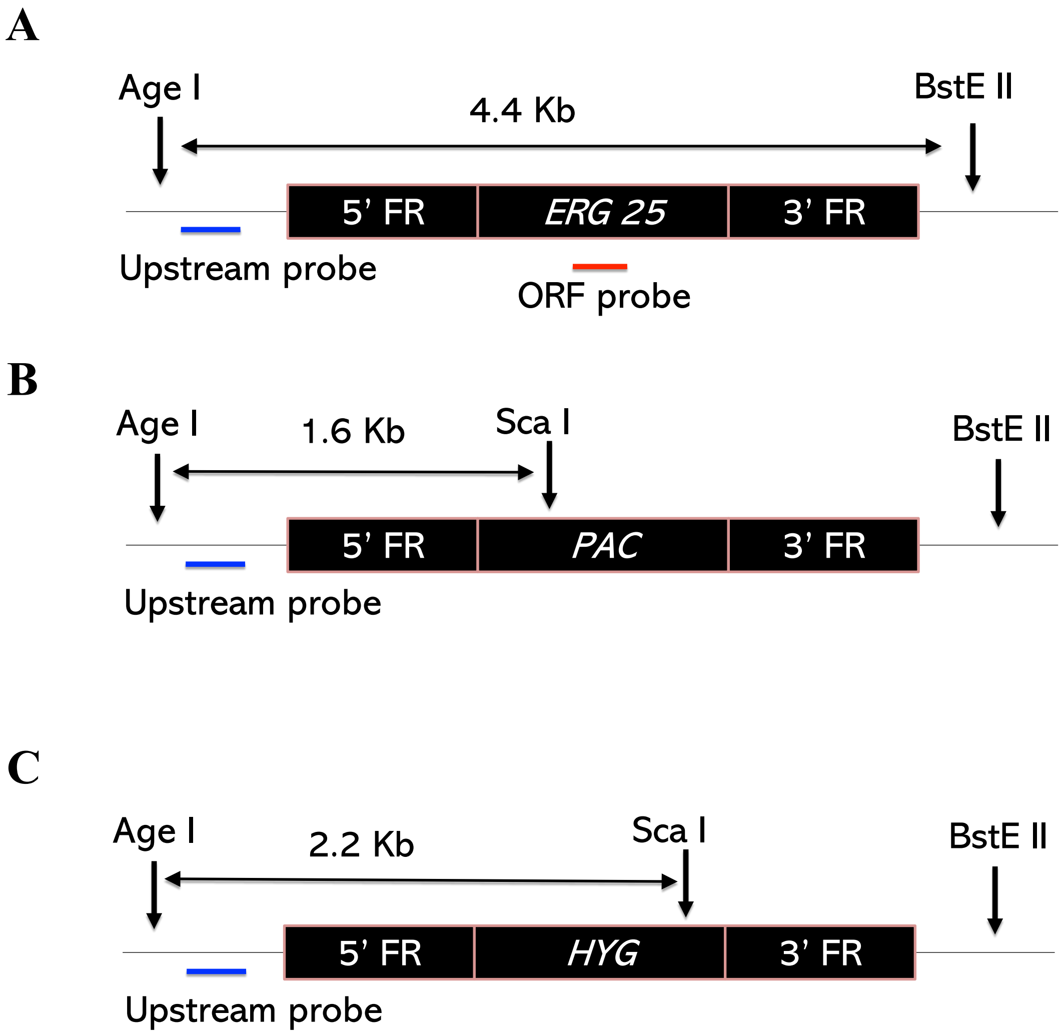

Figure S3. Schematic depiction of the Southern blot for *L. major ERG 25*. (A) The genomic locus of *ERG25* in WT *L. major* showing its open reading frame (ORF), 5' and 3' flanking regions (FR), recognition sites of Age I, BseE II and Sca I, and the positions of ORF probe and upstream probe. **(B-C)** Replacement of *ERG25* ORF with hygromycin (HYG) and puromycin (PAC) resistance genes. The expected sizes of DNA fragments recognized by the ORF probe or upstream probe were indicated.

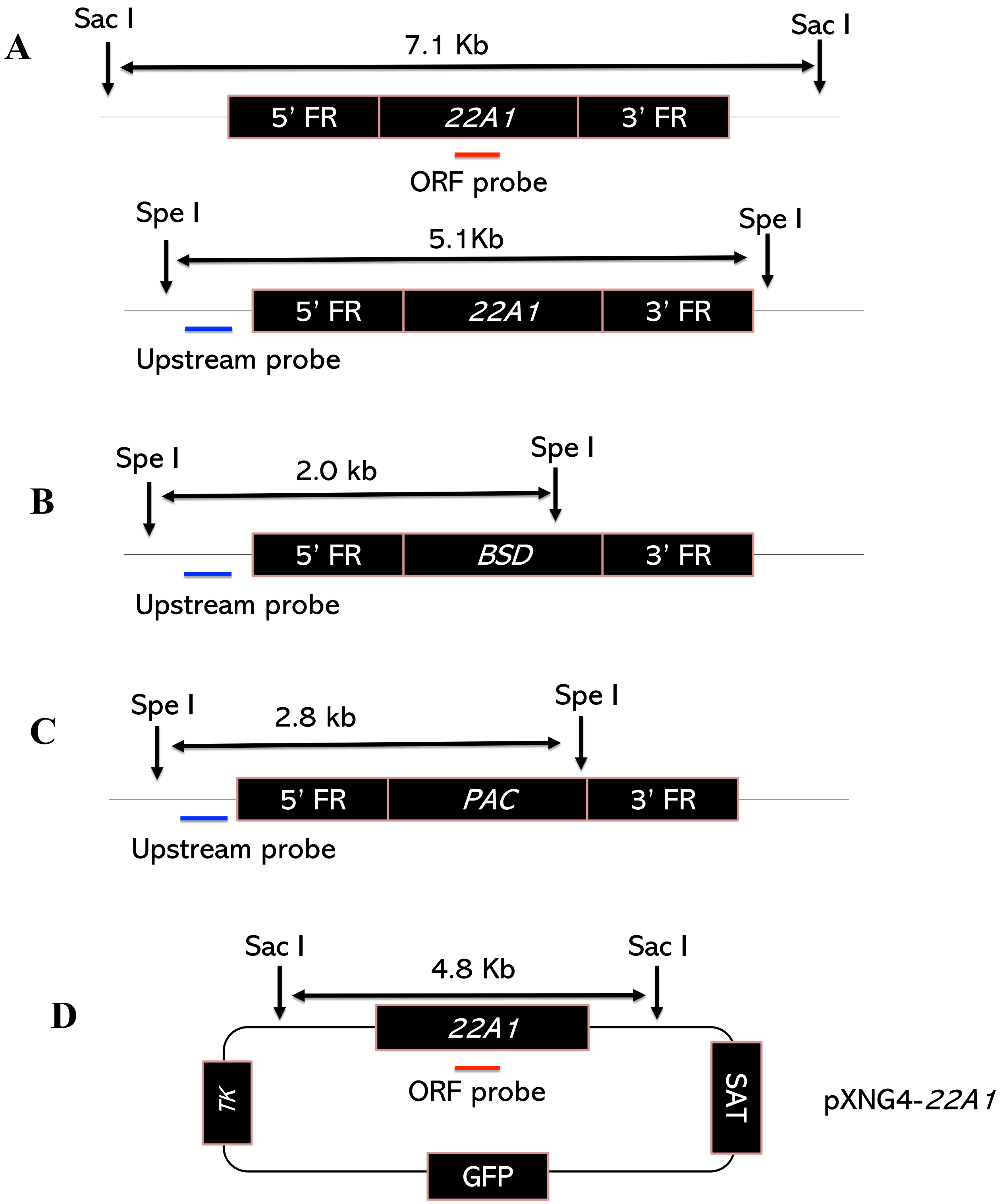

Figure S4. Schematic depiction of the Southern blot for *L. major CYP5122A1* (22A1). (**A**) The genomic locus of *CYP5122A1* in WT *L. major* showing its open reading frame (ORF), 5' and 3' flanking regions (FR), recognition sites of Sac I and Spe I, and the positions of ORF probe and upstream probe. (**B-C**) Replacement of 22A1 ORF with blasticidin (BSD) and puromycin (PAC) resistance genes. (**D**) Episomal expression of 22A1 from pXNG4-5122A1. TK: thymidine kinase, GFP: green fluorescent protein, SAT: nourseothricin resistance gene. The expected sizes of DNA fragments recognized by the ORF probe or upstream probe were indicated.

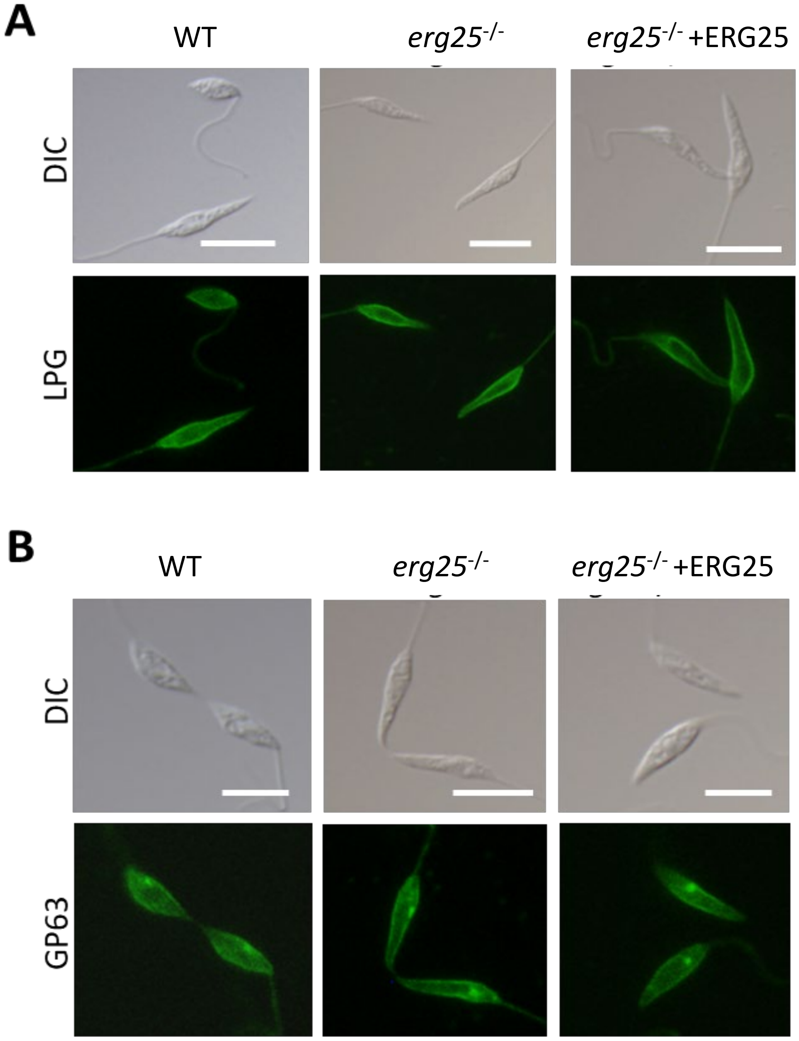

Figure S5. The expression of LPG and GP63 is not affected in *erg25$^{-/-}$* promastigotes. Log phase promastigotes were labeled with mouse anti-LPG (**A**) or anti-GP63 (**B**) monoclonal antibody followed by goat anti-mouse IgG-FITC. Scale bars: 10 µm.

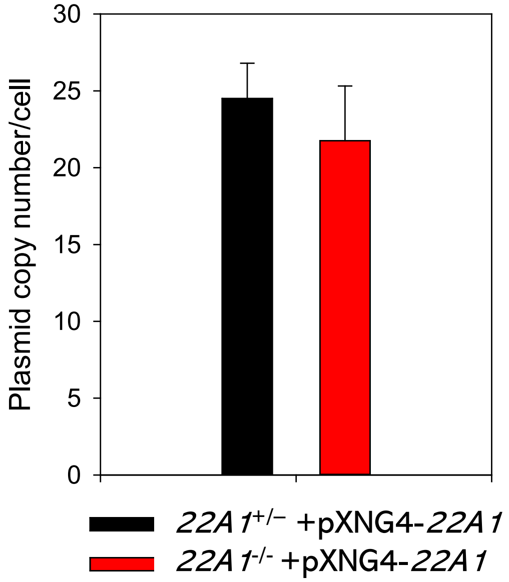

Figure S6. Plasmid levels in promastigotes. Stationary phase promastigotes of *22A1*$^{+/–}$ +pXNG4-*22A1* and *22A1*$^{-/-}$ +pXNG4-*22A1* were cultivated in the presence of SAT and their DNA was extracted to determine the average pXNG4-Lm22A1 plasmid copy numbers per cell by qPCR.

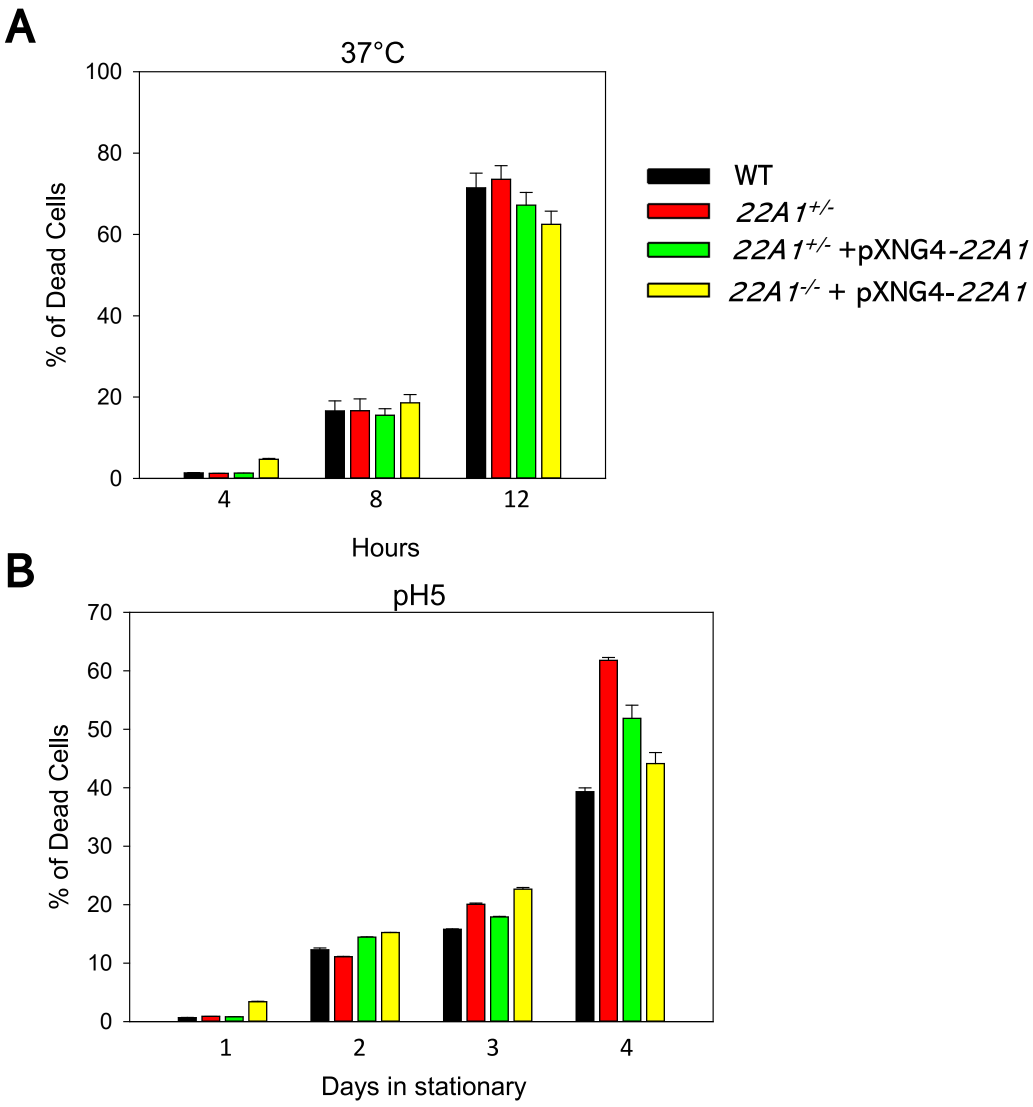

Figure S7. Stress response of *22A1* mutants. Day 1 stationary phase promastigotes were incubated in complete M199 medium (**A**:, pH7.4, 37 °C. **B**: pH5.0, 27 °C) and percentages of dead cells were determined by flow cytometry at the indicated times.. Error bars represent standard deviations from three experiments.

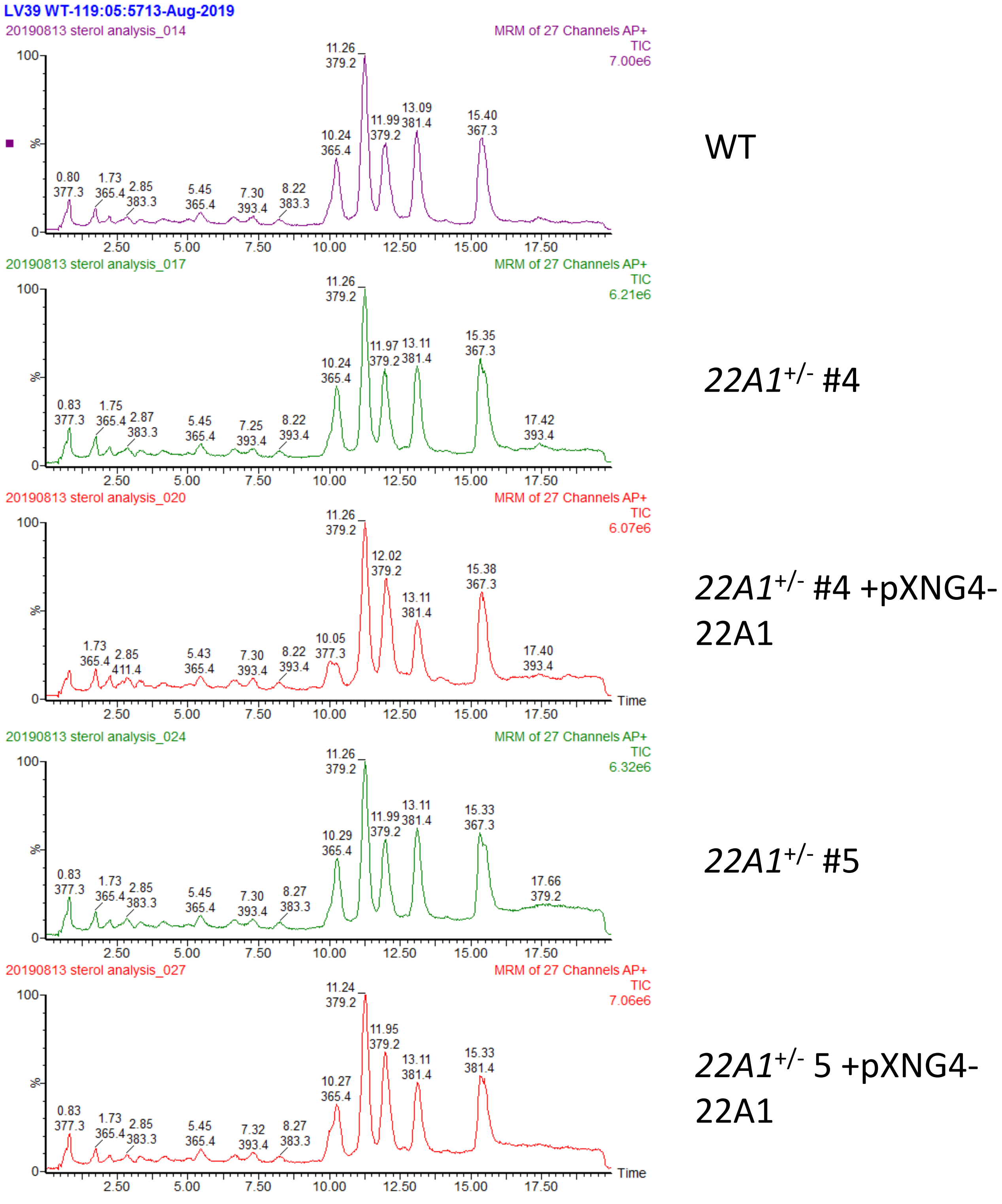

Figure S8. 22A1 half knockout and overexpression do not affect bulk sterol composition in *L. major*. Partial LC-MS chromatograms of free sterols from WT, *22A1*$^{+/-}$ *#4, 22A1*$^{+/-}$ *#4* +pXNG4-22A1, *22A1*$^{+/-}$ *#5,* and *22A1*$^{+/-}$ *#5* +pXNG4-22A1 promastigotes.

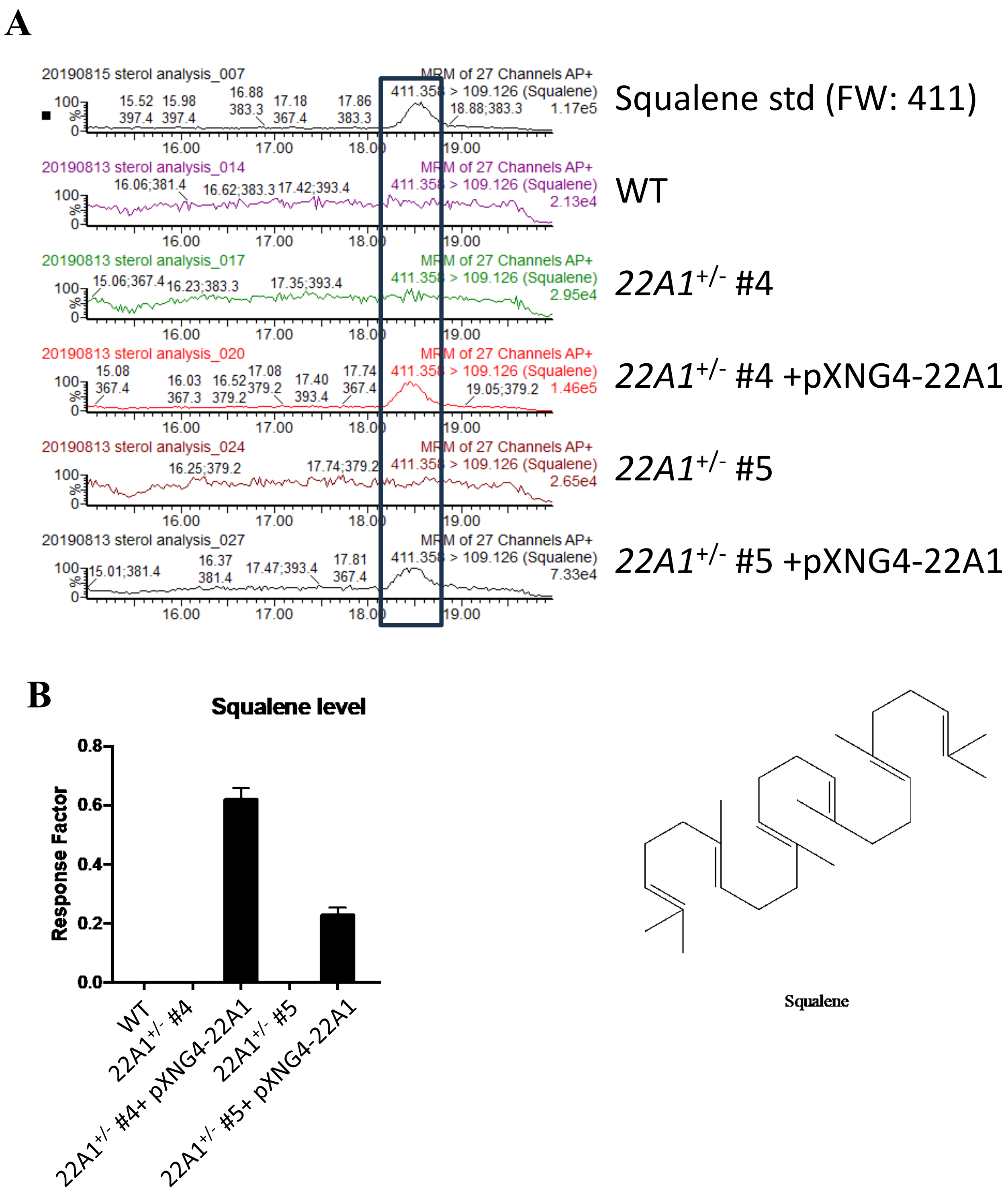

Figure S9. Squalene were detected in 22A1 overexpressing cells. (A) Partial GC chromatograms of total lipids from WT, *22A1$^{+/-}$ #4, 22A1$^{+/-}$ #4* +pXNG4-22A1, *22A1$^{+/-}$ #5,* and *22A1$^{+/-}$ #5* +pXNG4-22A1 promastigotes showed the presence of squalene in the boxed region. (**B**) The relative abundance of squalene was determined.

Table S1. List of oligonucleotides used in this study

Oligo #	Name	Purpose	Sequence
709	28s rRNA_forward	For qRT-PCR analysis of 28S rRNA	AAGATGGACCGGCCTCTAGT
710	28s rRNA_reverse	For qRT-PCR analysis of 28S rRNA	ATCCTTCCCCGCTCCAGTAT
359	5' ORF C4 methyl oxidase	To amplify the ERG25 ORF	GATCATagatct TCGATGGTGCTCGCGAAAAAGC
360	3' ORF C4 methyl oxidase	To amplify the ERG25 ORF	GATCATagatct CCTTCACGAACACGCGCGC
361	for 5' UTR C4 methyl oxidase	To amplify the 5'- flanking sequence of ERG25	GATCATgaattc TCAATGTGACTTGCGTTTAACTCCCTCC
362	rev 5' UTR C4 methyl oxidase	To amplify the 5'- flanking sequence of ERG25	GTCAGCggatccGATCTAactagt CGAGTGCCGAGGGATAAGGTGGAG
363	for 3' UTR C4 methyl oxidase	To amplify the 3'- flanking sequence of ERG25	GATCATggatcc AGGTGCTCAGGCACAAAACGACTTC
364	rev 3' UTR C4 methyl oxidase	To amplify the 3'- flanking sequence of ERG25	GATCATaagctt TCAACAGTTATGCGCATACATATTCACAG
602	Erg25_probe_F.P	To generate the ERG25 probe for Southern blot	GCGCACACCTTCAATGTG
603	Erg25_probe_R.P	To generate the ERG25 probe for Southern blot	CGAACACGACGTGTA
631	Erg25 probe out of 5'UTR F	To generate the ERG25 probe for Southern blot	CAGAGCTAAGTCGTGCTG
632	Erg25 probe out of 5'UTR R	To generate the ERG25 probe for Southern blot	GCTGTTCTGCGGATCATC
740	LmCYP-5'UTR-R	To amplify the 5'- flanking sequence of Lm22A1	GTC GCT actagt GTG AAA TGG GCG ACA AGA G
757	LmCYP-5'UTR-F	To amplify the 5'- flanking sequence of Lm22A1	CAT GCT gaattc CCT TTC TCT GTG CAC CCT TC
741	LmCYP-3'UTR-F	To amplify the 3'- flanking sequence of Lm22A1	CGG ACG actagt GGC TAG agatct AGC GAG CTG AGA ATT GCC
742	LmCYP-3'UTR-R	To amplify the 3'- flanking sequence of Lm22A1	GTC AAG aagctt GCA TTC ACG CAC GCT CTC
754	LmCYPORF-F	To amplify the Lm22A1 ORF	TCA GTA ggatcc ACC ATG GTG TTT GAC ACC GAC
755	LmCYPORF-R	To amplify the Lm22A1 ORF	GCG CT ggatcc TTACATGAACAACTTGTTCG
756	LmCYPORF-nostop-R	To amplify the Lm22A1-GFP fusion	GCG GTC gatatc CAT GAA CAA CTT GTT CGG
768	Lm22A1 ORF probe FP	To generate the Lm22A1 and Ld22A1 probe for Southern blot	GTC GCA TGC GAT GCG CAT C
769	Lm22A1 ORF probe RP	To generate the Lm22A1 and Ld22A1 probe for Southern blot	GTA GCG AGT CTG CAC GGT GC
770	Lm22A1 5'UTR probe FP	To generate the Lm22A1 probe for Southern blot	CAG TGA GCG AGA GAA CGA G
771	Lm22A1 5'UTR probe RP	To generate the Lm22A1 probe for Southern blot	CCT ACA TGA GCG TAG GAC AC

For Figure 2C (left)

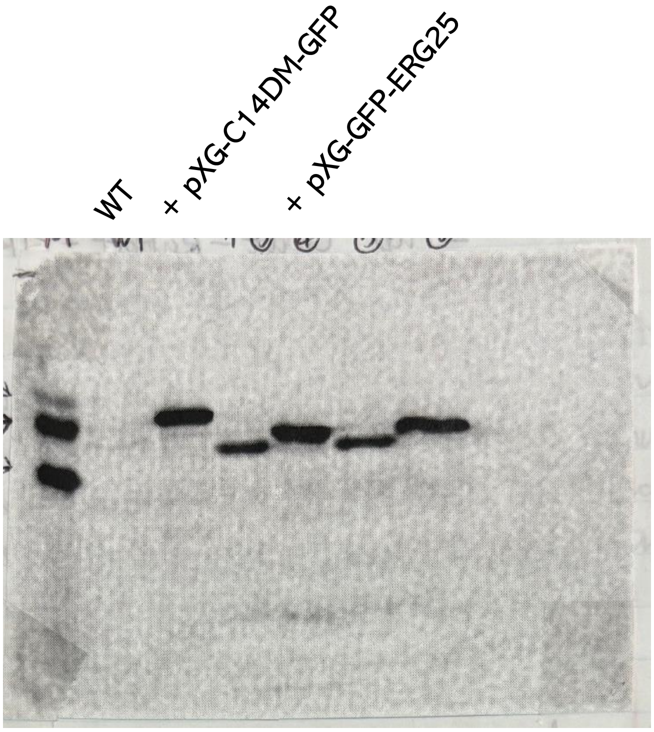

Anti-GFP

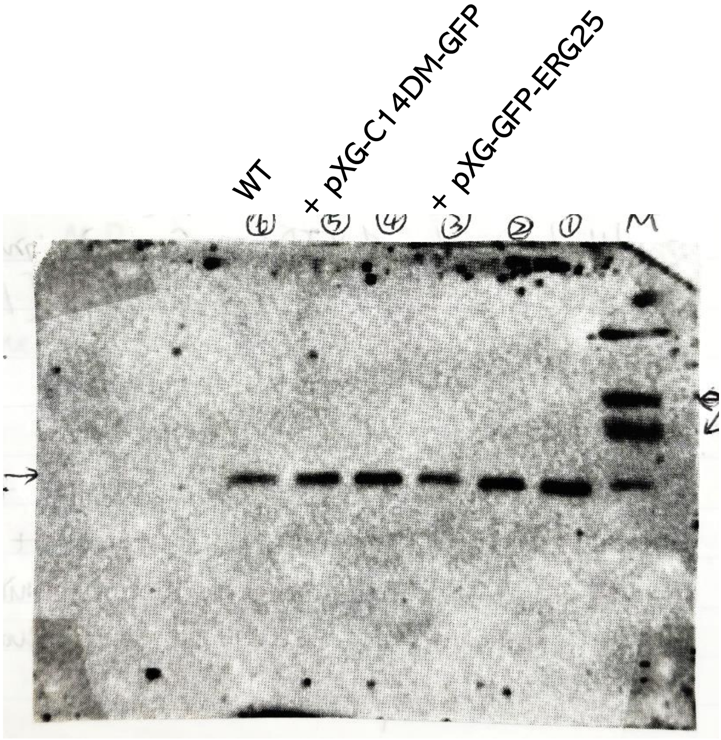

Anti-αtubulin

For Figure 2C (right)

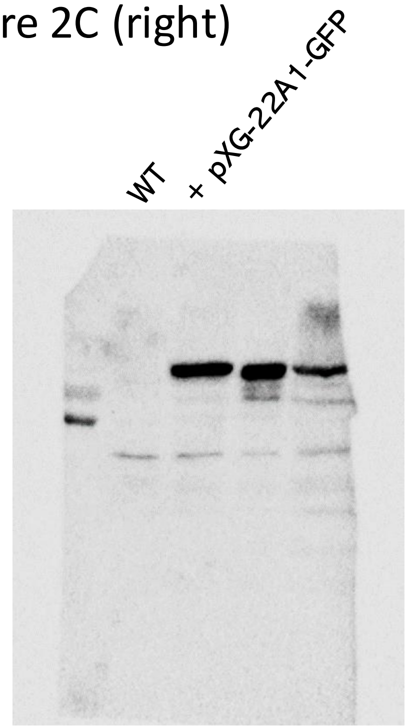

Anti-GFP

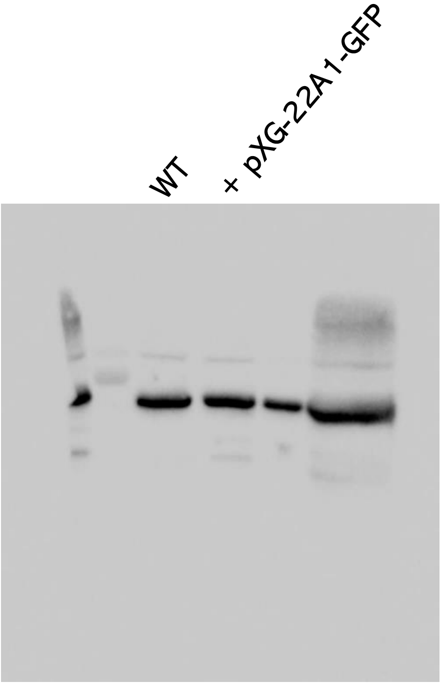

Anti-αtubulin

For Figure 3A

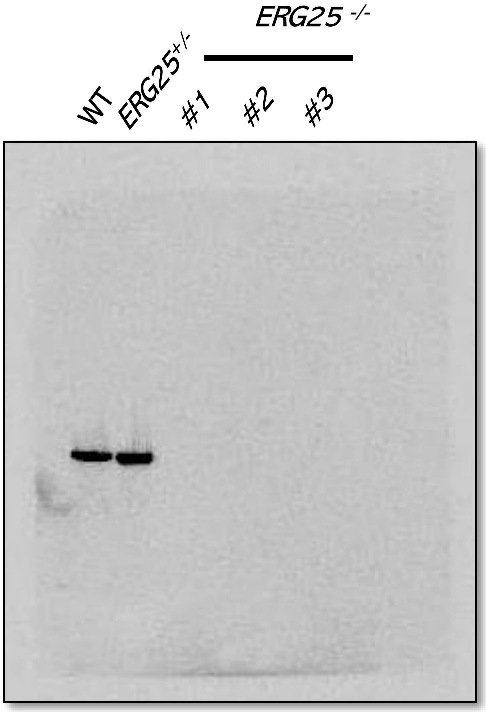

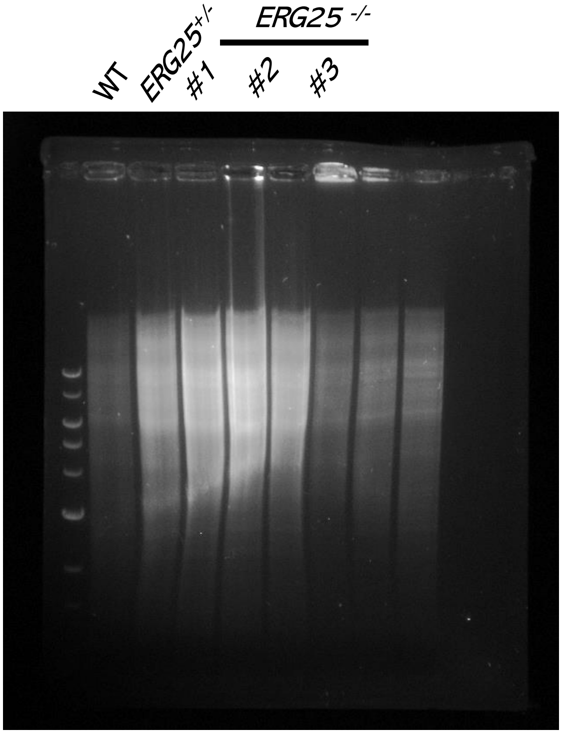

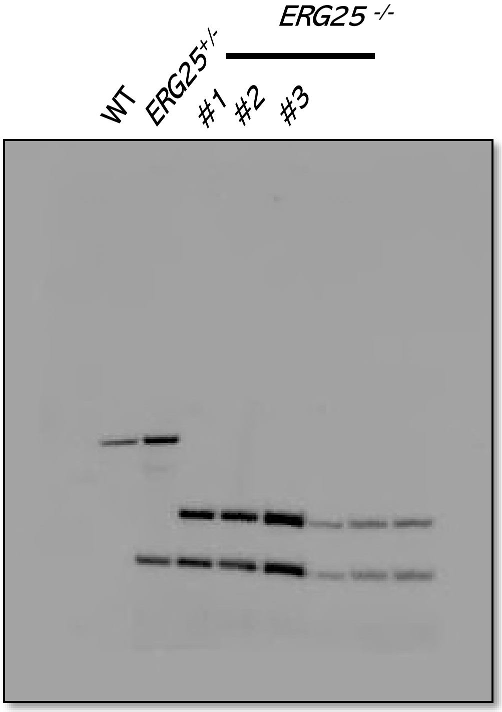

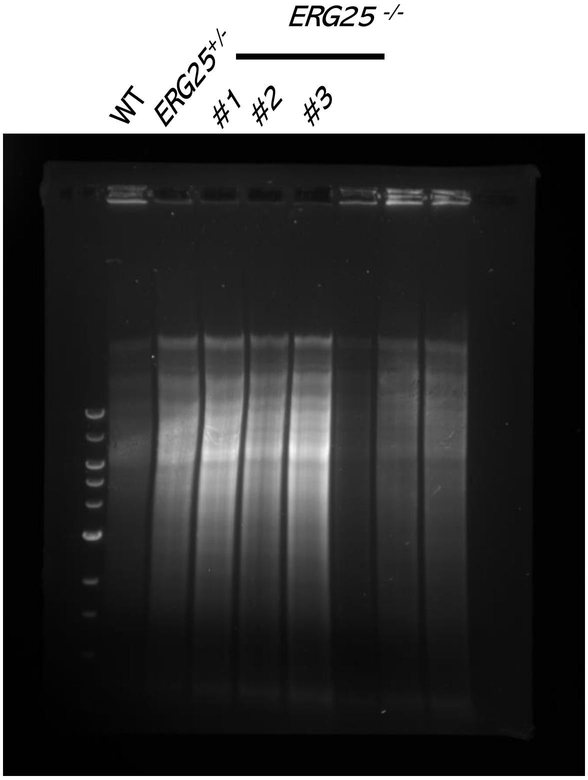

For Figure 3B

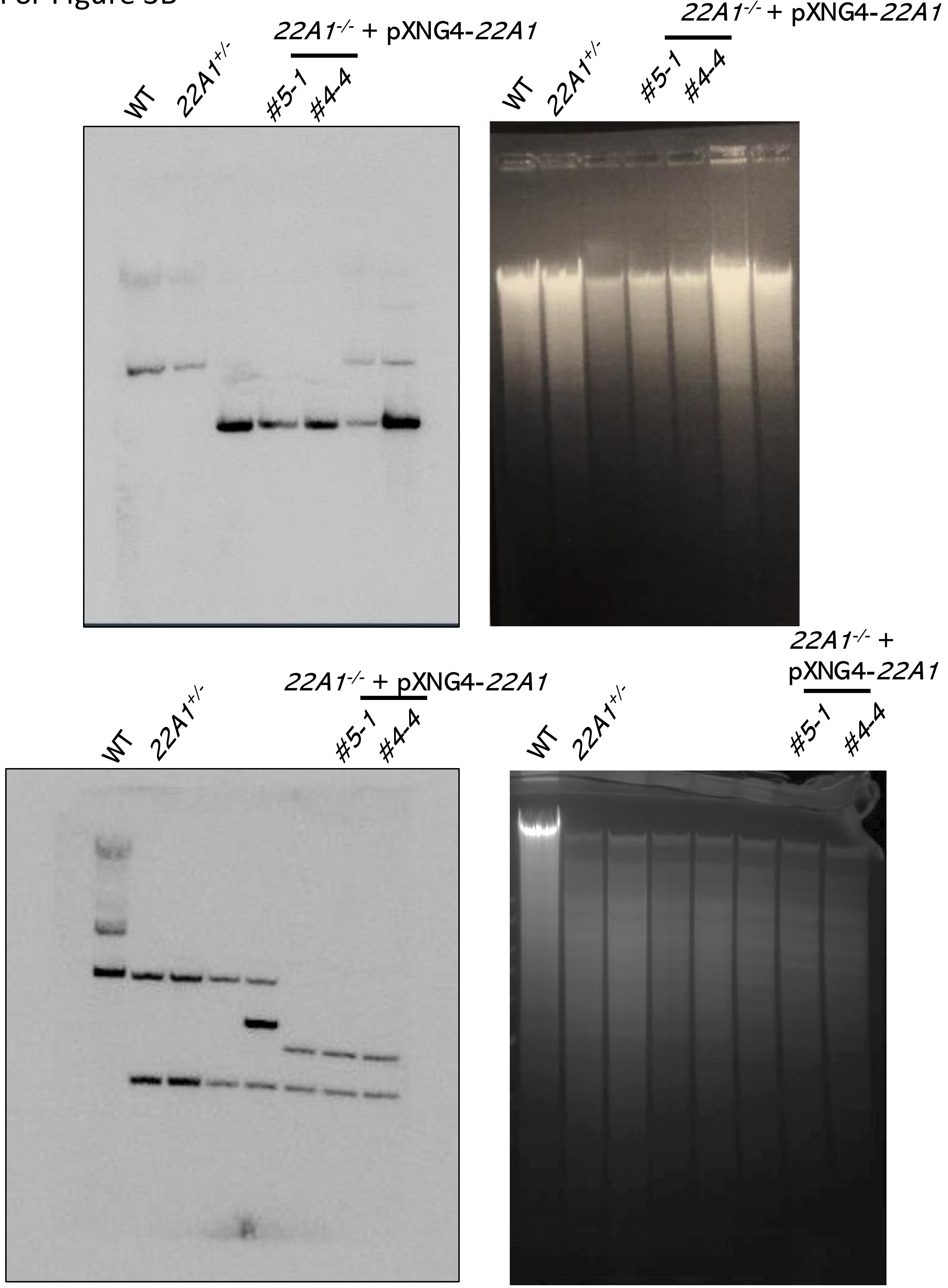

For Figure 9A (left)

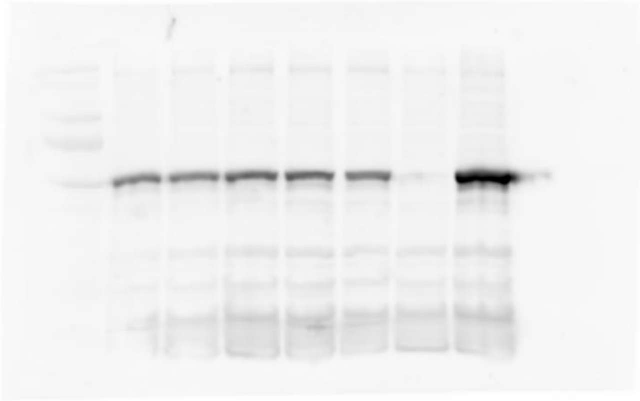

Anti-C14DM

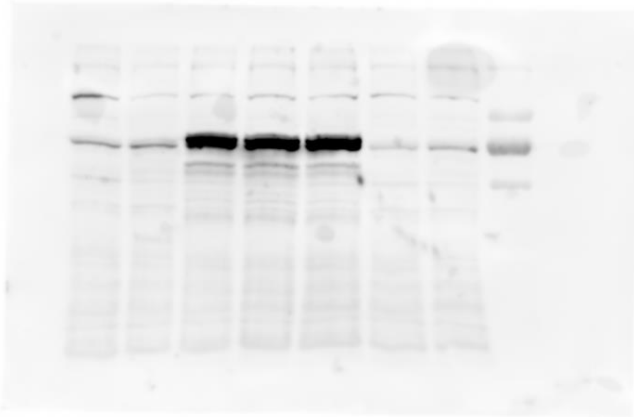

Anti-22A1

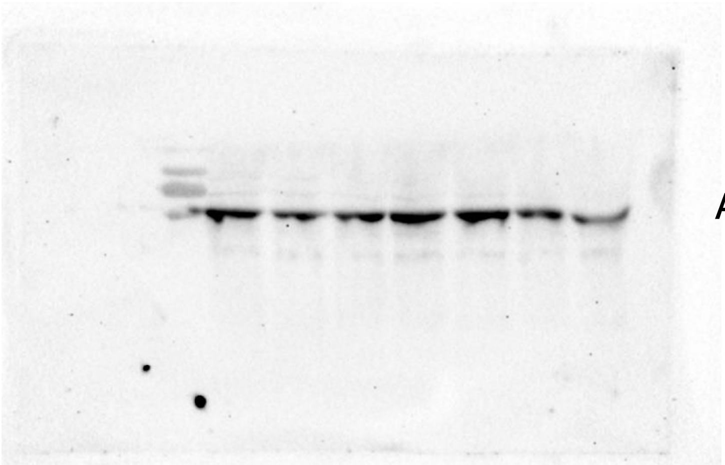

Anti-αtubulin

For Figure 9A (right)

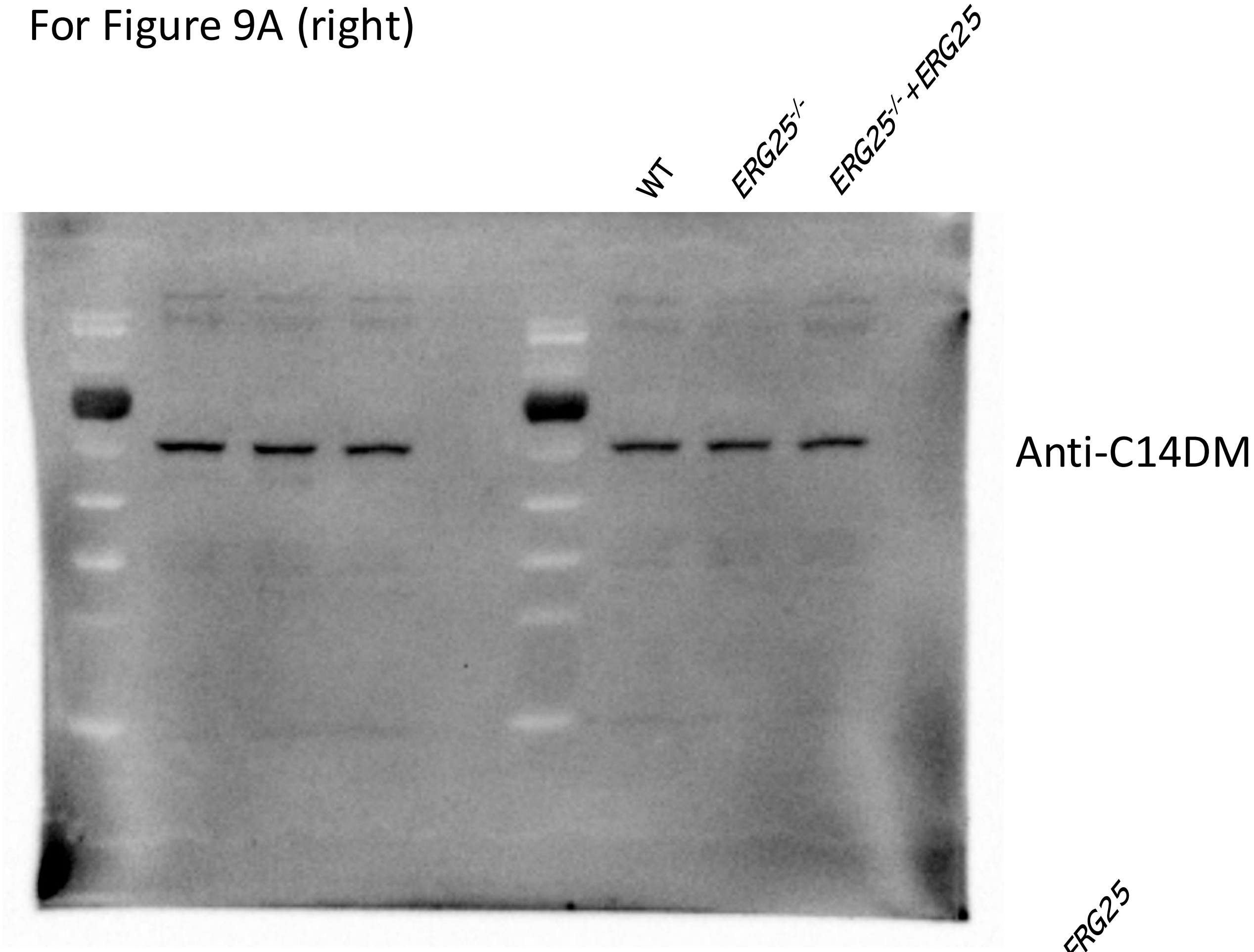

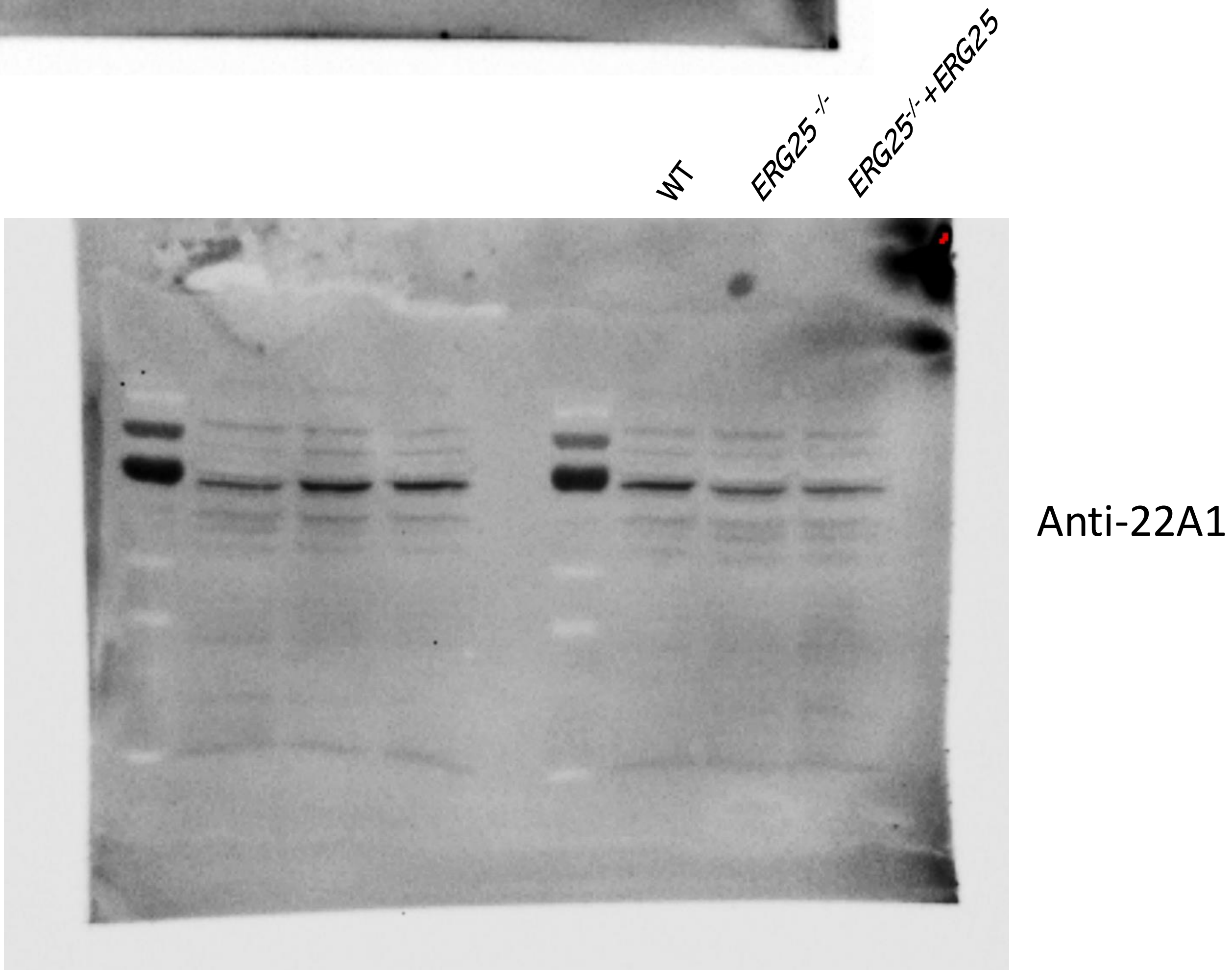

For Figure 9B (right)

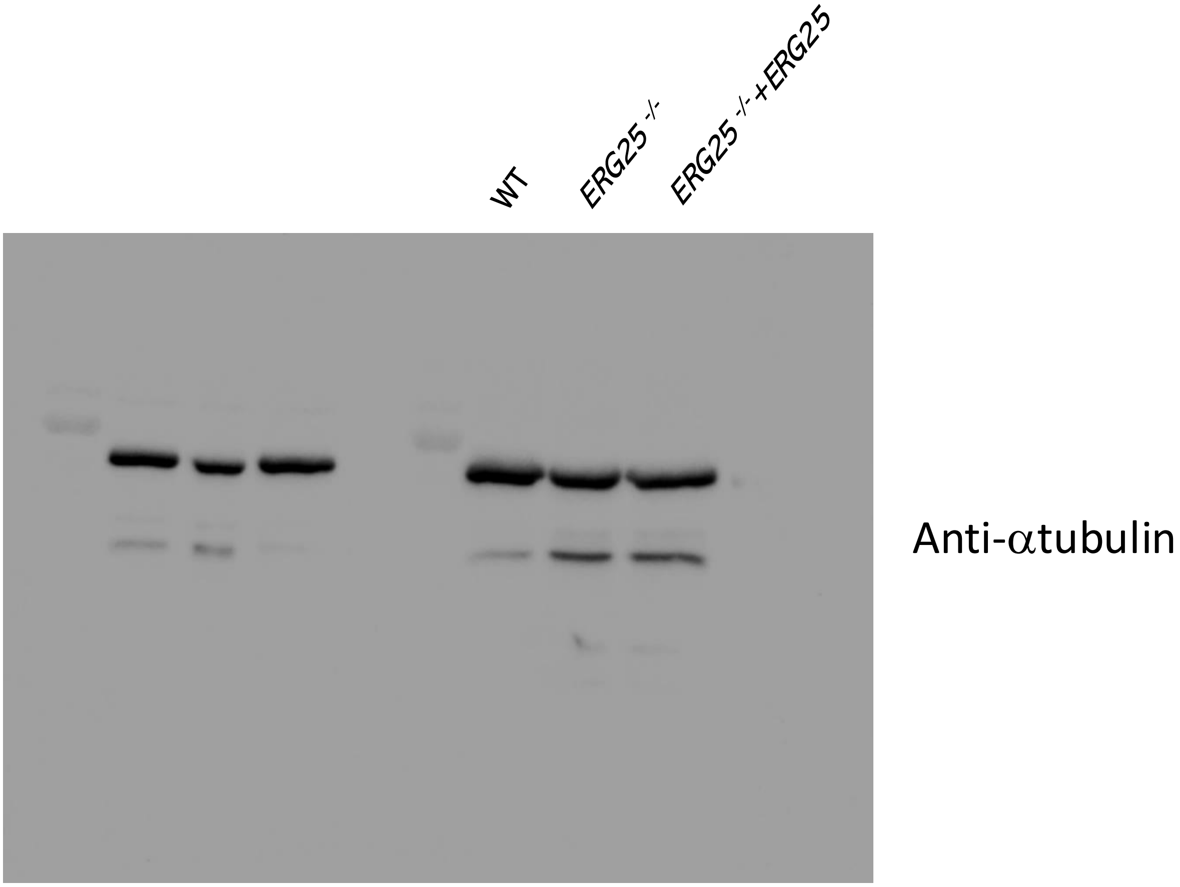

Supplement: Supplementary file 1 [file ijms-25-10908-s001.zip › ijms-3163554-supplementary.pdf]
